# The Effects of a Technology‐Assisted Hybrid Cardiac Rehabilitation (TecHCR) Program for Adults With Coronary Heart Disease: A Randomized Controlled Trial

**DOI:** 10.1111/wvn.70092

**Published:** 2025-12-28

**Authors:** Mei Sin Chong, Janet Wing Hung Sit, Kai Chow Choi, Anwar Suhaimi, Ying Jiang, Sek Ying Chair

**Affiliations:** ^1^ The Nethersole School of Nursing, Faculty of Medicine The Chinese University of Hong Kong Hong Kong SAR China; ^2^ Alice Lee Centre for Nursing Studies, Yong Loo Lin School of Medicine National University of Singapore Singapore; ^3^ Department of Rehabilitation Medicine Universiti Malaya Kuala Lumpur Malaysia

**Keywords:** cardiac rehabilitation, coronary disease, exercise, health belief model, health‐promoting behavior, technology

## Abstract

**Background:**

Technology‐assisted interventions offer a promising alternative to conventional cardiac rehabilitation. However, there is limited evidence on their effectiveness, particularly in non‐Western settings with emphasis on exercise self‐efficacy.

**Aims:**

To evaluate the effects of a 12‐week, technology‐assisted hybrid cardiac rehabilitation (TecHCR) program on physical, physiological, and psychological outcomes of patients with coronary heart disease.

**Methods:**

A two‐arm parallel randomized controlled trial including 160 participants was randomly assigned to either TecHCR or usual care. TecHCR was underpinned by the Health Belief Model, consisting of three supervised exercise training and occupational therapy sessions, a fitness watch for exercise self‐monitoring, six audio‐visual educational videos, and a weekly video call follow‐up. Data were collected at baseline, immediately post‐intervention, and at 24 weeks post‐intervention.

**Results:**

Participants in TecHCR demonstrated significantly greater improvement in exercise self‐efficacy (*β* = 5.909, 95% CI [3.146, 8.672]; *p* < 0.001), health‐promoting behaviors (*β* = 9.058, 95% CI [5.524, 12.591]; *p* < 0.001), and perceived anxiety levels (*β* = −1.255, 95% CI [−1.893, −0.616]; *p* < 0.001) at immediate post‐intervention and (*β* = 8.506, 95% CI [4.951, 12.061]; *p* < 0.001, *β* = 14.563, 95% CI [8.809, 20.317]; *p* < 0.001, *β* = −1.145, 95% CI [−1.975, −0.315]; *p* = 0.007, respectively) 24 weeks post‐intervention when compared with the control group. No statistically significant improvements were observed in perceived depression and cardiovascular risk factors.

**Linking Evidence to Action:**

The TecHCR program, combining supervised sessions with technology‐assisted components, is an effective approach for significantly improving exercise self‐efficacy, health‐promoting behaviors, and anxiety in patients with coronary heart disease. Healthcare institutions should consider implementing hybrid programs to overcome barriers to traditional cardiac rehabilitation, leveraging technology to extend support and maintain patient engagement beyond supervised sessions.

**Trial Registration:**

clinicaltrials.gov identifier: NCT04862351

## 1 Introduction

Coronary heart disease (CHD) remains a significant public health challenge despite advancements in healthcare systems. The 2024 Heart Disease and Stroke Statistics reveal that nearly half (48.6%) of adults aged 20 and over in the United States live with cardiovascular disease, with 7.1% diagnosed with CHD (Martin et al. 2024). Globally, 126.5 million adults with CHD accounted for 8.9 million deaths in 2017 (Dai et al. 2022). CHD affects not only physical (Li et al. 2021) and mental health (Palacios et al. 2018) but also imposes substantial economic burdens on society (Gordois et al. 2016; Savira et al. 2021). In 2021, CHD cost European Union health and social care systems 30 billion and was responsible for nearly half a million lost working years due to mortality (Luengo‐Fernandez et al. 2023). Based on a recent review of the economic costs of CHD across 22 countries, the pooled direct annual cost per patient amounted to as much as 137.8% of the gross domestic product (GDP) (Shakya et al. 2025). Given these detrimental effects of CHD, there is an increased emphasis on secondary prevention. Cardiac rehabilitation (CR) plays a pivotal role in providing comprehensive interventions for individuals with CHD, comprising exercise training, risk factor modification, patient education, nutritional counseling, and psychosocial management (Leon et al. 2005; Mehra et al. 2020). Exercise training, a central component of CR significantly lowers the risk of cardiovascular mortality and reduces hospital admission rates (Anderson et al. 2016). Additionally, CR has been shown to markedly improve health‐related quality of life (Chair et al. 2013; Dibben et al. 2023). Despite these benefits, CR utilization worldwide falls short of expectation, revealing that only 9% of patients with CHD participated in CR (McEvoy et al. 2024).

Various barriers to CR have been identified over the years, including inadequate availability (Ragupathi et al. 2017), time constraints (Wong et al. 2020), a lack of clear referral pathways (Clark et al. 2013), leading to long waiting periods from discharge to enrolment, and lack of interest or perceived needs (Sérvio et al. 2019; Wong et al. 2020). In response to these challenges, CR programs have evolved over the past decades from exercise‐based approaches to multi‐component strategies. During the COVID‐19 pandemic, CR was impacted due to its non‐emergency care nature. However, the pandemic also created a push factor to accelerate the integration of technology into CR. Technology‐assisted interventions, using information communication technologies such as the Internet, digital devices, and even landline telephones (Chong et al. 2021), can serve as alternatives to conventional, center‐based CR programs, offering comparable effectiveness on patient outcomes (Owen and O'Carroll 2024). For instance, Widmer et al. (2017) found that online and smartphone‐based CR significantly reduced weight in patients compared to standard care, but there were no significant differences in cardiovascular‐related rehospitalizations and emergency department visits between intervention and control groups. Similarly, participants engaging in group exercise training sessions through videoconferencing demonstrated higher adherence to the study protocol compared to center‐based CR, despite no significant differences being observed in physical outcomes and quality of life (Hwang et al. 2017).

Previous reviews predominantly included studies conducted in Western countries (Chong et al. 2021; Luijk et al. 2024), and if they included studies from Asia, they were primarily from China (Wongvibulsin et al. 2021). Identified gaps in technology‐assisted interventions include a lack of rigorous randomized controlled trials, theoretical frameworks, and emphasis on self‐efficacy, which is a vital mechanism for promoting self‐management among patients with chronic diseases (Chong et al. 2021).

A theoretical framework is essential for designing effective interventions (Graham et al. 2020), as poor theory application can limit intervention effectiveness (Timlin et al. 2020). The Health Belief Model (HBM), developed by psychologists in the 1950s, is instrumental in understanding how individuals change and maintain health behaviors (Champion and Skinner 2008). Initially, it comprised five constructs, including “perceived susceptibility,” “perceived severity,” “perceived benefits,” “perceived barriers,” and “cues to action,” and later “self‐efficacy” was added to the model to emphasize the role of self‐belief in overcoming challenges and maintaining behavioral change (Rosenstock et al. 1988). In CR, “perceived susceptibility” benefits patients as individuals are more likely to engage in recommended actions if they feel susceptible to a condition with serious consequences (Green et al. 2020). It is also important to emphasize the severity of CHD, as Redfern et al. (2007) found that a substantial number of patients (70%) had low awareness of their modifiable cardiovascular risk factors. Jones et al. (2014) highlight that “perceived susceptibility,” “perceived severity,” and “cues to action” contribute to an individual's threat perception. Furthermore, one of the crucial pieces of information for patients is the potential benefits of CR (Clark et al. 2013), and both perceived benefits and perceived barriers influence the likelihood of individuals adopting health behaviors (Jones et al. 2014). The addition of “self‐efficacy” as a construct is particularly relevant to exercise behavior during and after CR (Rodgers et al. 2013). Klompstra et al. (2018) point out that exercise self‐efficacy influences physical activity levels, as even high motivation may not result in increased physical activity if self‐efficacy is low. This underscores the importance of incorporating self‐efficacy during intervention development and evaluating exercise self‐efficacy as an outcome. Thus, the constructs of HBM can potentially enhance patient outcomes in CR.

The present study aimed to evaluate the effects of a technology‐assisted hybrid cardiac rehabilitation (TecHCR) program on physical, physiological, and psychological outcomes of patients with CHD. Given the importance of exercise self‐efficacy, we hypothesized that the TecHCR program would significantly improve the exercise self‐efficacy among patients with CHD at immediate post‐intervention and at 24 weeks post‐intervention compared with those receiving center‐based CR (usual care). We also hypothesized that the TecHCR program would lead to (1) better health‐promoting behaviors, (2) improved exercise capacity, (3) improved psychological well‐being, and (4) improved cardiovascular risk factors at immediate post‐intervention and 24 weeks post‐intervention.

## 2 Methods

This study has been reported in accordance with the Consolidated Standards of Reporting Trials (CONSORT) (Hopewell et al. 2025) and the Template for Intervention Description and Replication guidelines (Supporting Information: Appendices S1, S2).

### 2.1 Study Design

The TecHCR study used a two‐arm parallel randomized controlled trial design. The protocol of this study was registered on clinicaltrials.gov on 21 April 2021. A feasibility study was conducted prior to this study and was reported in detail. (Chong et al. 2023).

### 2.2 Participants

Patients were recruited from a non‐invasive cardiology clinic at the Universiti Malaya Medical Centre, the largest and oldest teaching hospital in Malaysia. This study was conducted between May 2021 and October 2023. In this setting, institutional protocol mandates the automatic referral of all inpatients with CHD to a phase II (outpatient) CR, commencing within 4–8 weeks post‐discharge. This protocol aligns with the strong recommendation from the 2011 American College for Cardiology Foundation/American Heart Association guideline for CR in eligible patients with post coronary artery bypass graft (CABG) surgery (Hillis et al. 2011). As a prerequisite for phase II CR, all patients must first undergo an exercise stress test for cardiovascular risk stratification (low, moderate, or high risk) (Abreu et al. 2018).

Eligible patients for this study were approached following the completion of the mandatory exercise stress test. Patients were eligible to participate in this study if they met the following criteria: aged 18 and over, diagnosed with CHD, underwent thrombolytic therapy or percutaneous coronary intervention or revascularization surgery, stratified as low or moderate risk for cardiac events based on the American Association of Cardiovascular and Pulmonary Rehabilitation (AACVPR) guidelines, owned a smart phone with Internet access, were able to converse in English or Malay, and had returned home after hospital discharge. Exclusion criteria included patients who were participating in other studies, had impaired hearing or vision, or were diagnosed with dementia, psychiatric illness (on active treatment), or pre‐existing mobility problems. A list of detailed eligibility criteria is available in the Supporting Information: Appendix S3. All participants provided written informed consent before the commencement of the study.

### 2.3 Sample Size

The idea of relying solely on the effect sizes reported in prior studies or results of small pilot studies is not considered appropriate for determining sample size (Association for Psychological Sciences 2016). The sample size for the study was powered to detect an effect size of Cohen's *d* = 0.5 on the primary outcome of exercise self‐efficacy, which is conventionally regarded as a medium effect size (Cohen 1992). Using the power analysis software G*Power 3.1 (Faul et al. 2009), it was estimated that a sample size of 64 participants per group was needed to detect an effect size of 0.5 with 80% power at a two‐sided 5% level of significance. Given an estimated attrition rate of 20%, a total of 160 participants, with 80 per group was recruited in this study.

### 2.4 Randomization, Allocation Concealment, and Blinding

In this study, participants were allocated to either the intervention or control groups in a 1:1 ratio using block randomization with varying block sizes of 4 and 6. This method ensured a balanced distribution in each block (Polit and Beck 2017) and kept the person who conducted the recruitment of participants blinded to the block sizes, minimizing the predictability in participant allocation (Efird 2011). The research assistant who enrolled participants did not have access to the random allocation sequence. An independent statistician prepared a computer‐generated random sequence using an online randomization program (http://www.randomization.com/). Participants were randomly assigned to either the intervention or control group using individual opaque sealed envelopes enclosing group identifiers based on the generated random sequence. Due to the pragmatic nature of the intervention, complete blinding was not feasible. Although participants were not informed of their assigned group, specific components of the intervention might have allowed them to deduce their allocation. Intervention providers could not be blinded to the treatment they were administering. Outcome assessors who were not directly involved in the study were blinded to participant group allocation. To maintain blinding, these assessors were not present during the intervention delivery.

### 2.5 Intervention

#### 2.5.1 Control Group

Participants assigned to the control group received a 12‐week standard care offered by the outpatient clinic, which included six or eight in‐person sessions of supervised exercise training and occupational therapy. Participants were encouraged to engage in at least 30 min of moderate‐intensity exercise on five or more days per week (at least 150 min/week) as recommended by the American Heart Association (Lane‐Cordova et al. 2022). The prescription for moderate‐intensity exercise aimed to achieve a heart rate range between 60% and 80% of the heart rate reserve (Chen et al. 2022). Both control and intervention groups were provided with a daily log on exercise and dietary practices to mitigate any psychological effects.

#### 2.5.2 Intervention Group

Participants in the intervention group received a 12‐week, technology‐assisted hybrid CR program led by a nurse (first author) who had substantial experience working in cardiac intensive care and coronary care units. Each participant was provided with a consumer‐based fitness watch, the Amazfit Band 5 after the baseline assessment. In this study, although the reliability and validity of the Amazfit Band 5 were not explicitly tested, its heart rate readings were compared with those reported on the treadmill during a face‐to‐face, supervised exercise training session conducted in Week 1. A recently published validation study by Ibrahim et al. (2024), assessed the heart rate accuracy and precision of older generations of Amazfit fitness watch models (Amazfit Cor and Amazfit Bip) between November 2018 and June 2019, which found that the mean absolute percentage error (MAPE) of the photoplethysmography was within 10% of the standard acceptable range established by the 12‐lead ECG measurements throughout the six stages of the modified Bruce exercise stress test. A MAPE within 10%, is regarded as reliable (Jo et al. 2016; Thiebaud et al. 2018).

Participants were briefed on how to use the watch and sync their exercise data at the end of each exercise session to the Zepp app, an existing health app. Thereafter, each participant attended three in‐person sessions, with each session consisting of 1‐h supervised exercise training led by a cardiac physiotherapist, followed by a 1‐h occupational therapy session conducted by an occupational therapist on the same day. Participants were required to independently exercise at moderate intensity, either at home or outdoors, on a regular basis according to the recommendation by the American Heart Association. Participants were advised on important exercise precautions, such as using the Rating of Perceived Exertion scale to gauge exercise intensity. Additionally, guidance was provided on recognizing situations that require immediate medical attention. Participants were instructed to wear their fitness watch throughout the day, especially while exercising. Exercise data were synced to the Zepp app and automatically transferred to UMFit, a website for remote monitoring by the research team. In addition, participants received a total of six audio‐visual educational videos covering topics such as CR, heart disease, exercise, diet, smoking cessation, and stress management. These videos were 10–15 min long and included voice‐over explanations (photos related to the exercise data, website, and educational videos are as shown in the Supporting Information: Appendix S4). Weekly follow‐up video calls led by the first author, each lasting about 20–30 min, were also scheduled for each participant. Table 1 presents the intervention development of TecHCR and the integration of the HBM. For example, the HBM construct of cues to action was integrated into asynchronous telemonitoring, serving as an external trigger for participants to independently perform the recommended duration of moderate‐intensity exercise, with the aim of enhancing their exercise self‐efficacy.

**TABLE 1:** TecHCR intervention development with the integration of Health Belief Model.

Week	0	1	2	3	4	5	6	7	8	9	10	11	12
Activity	Build rapport Demonstration and return demonstration for using the fitness watch and uploading the data	**At outpatient CR clinic** Face‐to‐face, individualized, supervised session (1 h exercise training by a cardiac physiotherapist and 1 h occupational therapy by an occupational therapist)		**At outpatient CR clinic** Face‐to‐face, individualized, supervised session (1 h exercise training by a cardiac physiotherapist and 1 h occupational therapy by an occupational therapist)									**At outpatient CR clinic** Face‐to‐face, individualized, supervised session (1 h exercise training by a cardiac physiotherapist and 1 h occupational therapy by an occupational therapist)
		**Asynchronous telemonitoring via wearable technology (fitness watch)** (Recommended to perform ≥ 150 min/week of moderate‐intensity exercise or ≥ 5 times/week [30 min each])—Based on Health Belief Model construct: Cues to action—The heart rate and exercise data on the fitness watch serves as the biofeedback strategy that will be used an external trigger											
		**Telecoaching** led by a nurse (first author) via Google Meet (20–30 min each session/week)—based on Health Belief Model constructs: (1) perceived benefits, (2) perceived barriers, (3) cues to action, and (4) self‐efficacy—To provide positive reinforcement to portray the positive benefits of adopting health behavior changes—To facilitate problem‐solving strategies in patients to overcome barriers in adopting health behavior changes—To provide feedback and advice as external cues for participants to activate the recommended health actions—To build participant's beliefs about own's ability to adopt health behavioral changes by using incremental setting strategies											
		**Audio‐visual educational video (1 video per week)** — Based on Health Belief Model constructs: (1) perceived susceptibility and severity and (2) perceived benefits — To provide educational support with accurate information of cardiovascular risk factors, consequences of the disease and recommended action. — To provide information on the benefits of recommended healthy lifestyle and diet						—					
		Daily log for exercise and diet											

### 2.6 Measurements

Sociodemographic (e.g., age, gender, ethnicity, marital status) and clinical data (e.g., medical history, documented procedure of PCI or CABG) of participants were collected at baseline (T0) using a self‐designed questionnaire. To provide a clearer overview of the entire sample, age was categorized into middle‐aged, 40–59 years (Lachman 2001) and ≥ 60 years (Chan et al. 2022).

#### 2.6.1 Primary Outcome

The primary outcome was participants' exercise self‐efficacy, which was assessed at three time points, including baseline (T0), immediate post intervention (T1), and 24 weeks post intervention (T2). To assess participants' perceived exercise self‐efficacy to consistently perform exercise routines, both the English and Malay versions of Bandura's 18‐item exercise self‐efficacy scale (ESE) (Bandura 2006) were used in this study. Each item in the English version is scored on a scale from 0 = *cannot do at all* to 100 = *highly certain can do*. This version demonstrated high internal consistency with a Cronbach's alpha of 0.95 in a CR setting (Everett et al. 2009). The Malay version also exhibited desirable psychometric properties (Sabo et al. 2019). In our study, the Cronbach's alpha was 0.959, indicating high internal consistency.

#### 2.6.2 Secondary Outcomes

The secondary outcomes were health‐promoting behaviors, exercise capacity, psychological well‐being, and cardiovascular risk factors, which were evaluated at the same time points as the primary outcome (T0, T1, and T2).

To assess participants' engagement in health‐promoting behaviors, this study used the 52‐item Health‐Promoting Lifestyle Profile II (HPLP II) (Walker et al. 1987). This measurement tool is comprised of 6 subscales: (1) health responsibility, (2) spiritual growth, (3) physical activity, (4) interpersonal relationships, (5) nutrition, and (6) stress management. It is a 4‐point response scale of 1 (*never*) to 4 (*routinely*). The mean score of each subscale was computed to preserve the 1–4 metric of item responses. The alpha coefficients for the subscales varied between 0.793 and 0.872 (Walker and Hill‐Polerecky 1996). In the Malay version of the HPLP II, all subscales demonstrated satisfactory construct reliability, with values between 0.737 and 0.878 (Kuan et al. 2019). In our study, the entire scale demonstrated high internal consistency, evidenced by a Cronbach's alpha of 0.957. Specifically, the Cronbach's alpha values are as follows, with health responsibility at 0.837, spiritual growth at 0.864, physical activity at 0.768; interpersonal relationships at 0.812, nutrition at 0.724, and stress management at 0.827.

The exercise capacity was measured using the exercise stress test with the modified Bruce protocol. According to Faselis et al. (2014), exercise capacity is assessed using metabolic equivalents (METs), where one MET is defined as 3.5 mL of O_2_/kg/min, representing the oxygen consumption rate when an individual is sitting at rest (Jetté et al. 1990). Participants had electrodes placed on their chests; their resting 12‐lead ECG, blood pressure and heart rate were obtained before the test. In the Bruce protocol, participants will undergo successive 3‐min stages, with each requiring them to walk faster on a steeper incline. However, the modified Bruce protocol is designed for patients who are unable to exercise vigorously, requiring less intense effort (Garner et al. 2017). The testing protocol was modified based on participants' tolerance, with the goal of achieving an exercise duration of 6–12 min. During the test, participants had continuous monitoring of 12‐lead ECG and heart rate, while their blood pressure was measured at regular intervals (at the end of each 3‐min stage). In this study, MET was automatically generated by the treadmill machine. A higher MET level signifies greater exercise capacity.

The psychological well‐being of participants was assessed using the 14‐item Hospital Anxiety and Depression Scale (HADS) (Zigmond and Snaith 1983). This scale consists of two subscales, anxiety and depression with seven items each using a 4‐point Likert scale ranging from 0 to 3. Both anxiety and depression total scores can range from 0 to 21; scores of ≤ 7 are deemed normal, between 8 and 11 suggest borderline, and scores > 11 indicate significant psychological morbidity (Zigmond and Snaith 1983). The Cronbach's alpha coefficients for anxiety and depression range between 0.68 and 0.93 and 0.67 and 0.90, respectively, both demonstrating satisfactory internal consistency. The Malay adaptation of HADS has shown adequate reliability, with Cronbach's alpha values of 0.88 for anxiety and 0.79 for depression (Yusoff et al. 2011). In our study, the Cronbach's alpha coefficients were 0.787 for anxiety and 0.754 for depression.

Cardiovascular risk factors including systolic and diastolic blood pressure, fasting lipid profile (HDL‐C, LDL‐C, TG, TC), glycated hemoglobin, body mass index (BMI), and waist circumference were assessed.

### 2.7 Ethical Considerations

Ethical approvals were obtained from The Joint Chinese University of Hong Kong—New Territories East Cluster Clinical Research Ethics Committee (The Joint CUHK‐NTEC CREC) (Reference Number: 2020.621) and UMMC Medical Research Ethics Committee (MREC) (Reference No. 202117‐9674). This study was conducted in accordance with the principles of the Declaration of Helsinki. All participants were informed about the study aims and objectives, its procedure, and its voluntary nature. Informed written consent was obtained from all participants before the baseline data collection.

### 2.8 Statistical Analysis

Data analyses were performed using the IBM SPSS 26 (IBM Corp. Armonk, NY). Descriptive statistics such as mean, standard deviation, range, frequency, and percentage were used to summarize participants' sociodemographic and clinical data. The analysis of baseline comparability for both intervention and control groups was conducted using inferential statistics. Skewness and kurtosis statistics and normal Q–Q plots were used to assess the normality of continuous data distribution. For continuous variables following a normal distribution, independent *t*‐tests were employed, while the Mann–Whitney *U* test, a nonparametric test was used for variables that were not normally distributed. The chi‐square and Fisher's exact tests were performed to compare categorical variables between two groups. The differential changes in each outcome across the time points between groups were compared using the generalized estimating equation (GEE) model in accordance with the intention‐to‐treat (ITT) principle. According to McNeish and Stapleton (2016), the GEE model leverages a quasi‐likelihood approach to accommodate missing data, provided the sample size is not too small, and the data are missing completely at random (MCAR). In this study, Little's MCAR test was performed, yielding a chi‐square = 281.907, df = 255 Sig. = 0.119, suggesting that the data were missing completely at random. All tests were 2‐sided, and a *p*‐value < 0.05 was considered statistically significant.

## 3 Results

**FIGURE 1:**
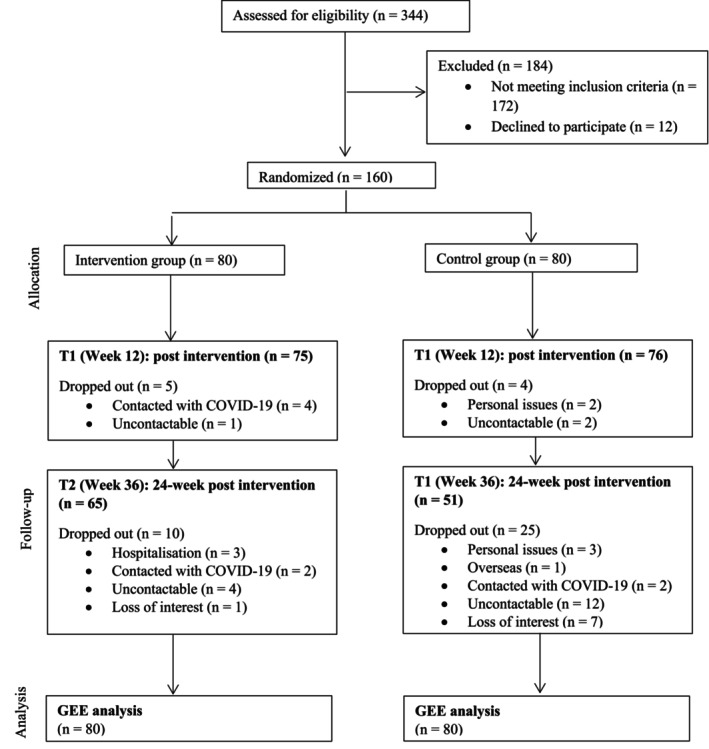
Flow diagram on recruitment and retention of participants.

A total of 160 participants were randomized to receive either TecHCR (intervention group, *n* = 80) or usual care (control group, *n* = 80). The recruitment and retention of participants throughout the study are detailed in the CONSORT flow diagram (Figure 1). Overall, 44 participants (27.5%) did not complete the study, with 15 from the intervention group and 29 from the control group. In the TecHCR group, five dropouts occurred at the T1 assessment with reasons such as being contacted with COVID‐19 infection (*n* = 4) and being uncontactable (*n* = 1). At the T2 assessment (24‐week post‐intervention), a further 10 participants were lost due to being uncontactable (*n* = 4), hospitalization (myocarditis, broken sternal wire, and spinal cord compression) (*n* = 3), COVID‐19 infection (*n* = 2), loss of interest (*n* = 1). In the control group, four dropouts occurred in T1 due to personal issues (*n* = 2) and being uncontactable (*n* = 2). At T2, 25 participants were lost due to reasons such as being uncontactable (*n* = 12), loss of interest (*n* = 7), personal issues (*n* = 3), COVID‐19 infection (*n* = 2), and moving overseas (*n* = 1). TecHCR was delivered as intended by a team of healthcare providers (including cardiac physiotherapists and occupational therapists), led by a nurse (first author). No harms or unintended events were reported in both the intervention and control groups.

### 3.1 Baseline Characteristics

Baseline sociodemographic and clinical characteristics were generally comparable between intervention and control groups (Table 2). The age ranged between 33 and 76 years old, 51.9% were in their middle adulthood and only 8.1% of participants aged below 40 years old. The majority (95.0%) were male, Malays (59.4%), married (92.5%), and 96.2% had attained a secondary school education or above. A total of 55% participants were non‐smokers. More than half of the participants (81.3%) had low risk for cardiac events, 77.5% of them had a myocardial infarction before being referred to the outpatient CR program. The majority (67.5%) underwent PCI and nearly 32% had surgical revascularization through CABG. The most prevalent comorbidity among participants was dyslipidemia (92.5%). All participants were on antiplatelet and statin medications. Seven variables were identified as covariates, based on a *p*‐value < 0.25 and existing literature (Barkley and Fahrenwald 2013; Song and Lee 2001). These variables included age, education, income, AACVPR risk category, BMI, dyslipidemia, and diabetes, which were adjusted in the GEE analysis to enhance the precision of the effect estimates for the intervention.

**TABLE 2:** Sociodemographic and clinical characteristics of participants (*N* = 160).

Characteristics	Total (*N* = 160) M ± SD/*n* (%)	Intervention (*n* = 80) M ± SD/*n* (%)	Control (*n* = 80) M ± SD/*n* (%)	*X* ^2^/*t*	*p*
Age group (years) (range:33–76)				4.133	0.136^a^
< 40	13 (8.1)	10 (12.5)	3 (3.8)		
40–59	83 (51.9)	39 (48.7)	44 (55.0)		
≥ 60	64 (40.0)	31 (38.8)	33 (41.2)		
Gender					0.276^b^
Male	152 (95.0)	74 (92.5)	78 (97.5)		
Female	8 (5)	6 (7.5)	2 (2.5)		
Ethnicity				2.883	0.406^b^
Malay	95 (59.4)	43 (53.2)	52 (65.0)		
Chinese	34 (21.2)	18 (22.5)	16 (20.0)		
Indian	29 (18.1)	18 (22.5)	11 (13.7)		
Others	2 (1.3)	1 (1.3)	1 (1.3)		
Marital status				0.461	1.000^b^
Married	148 (92.5)	74 (92.5)	74 (92.5)		
Single	5 (3.1)	3 (3.8)	2 (2.5)		
Divorced/widowed	7 (4.4)	3 (3.8)	4 (5.0)		
Education				5.43	0.066^b^
Primary	6 (3.8)	2 (2.5)	4 (5.0)		
Secondary	60 (37.5)	37 (46.2)	23 (28.7)		
Tertiary	94 (58.8)	41 (51.3)	53 (66.3)		
Occupation				0.027	1.000
Employed	103 (64.4)	52 (65.0)	51 (63.7)		
Retired/unemployed/homemaker	57 (35.6)	28 (35.0)	29 (36.3)		
Monthly household income (RM)				1.234	0.341
< 5000 (< US$ 1220)	73 (45.6)	33 (41.2)	40 (50.0)		
≥ 5000 (≥ US$ 1220)	87 (54.4)	47 (58.8)	40 (50.0)		
Driving	121 (75.6)	63 (78.7)	58 (72.5)	0.848	0.462
Distance from home to CR (km) median, IQR^d^	18 (13.0–26.4)	17 (13.3–28.8)	19.0 (12.3–25.4)		0.615^c^
Smoking status				1.71	0.495
Never	88 (55.0)	45 (56.3)	43 (53.8)		
Former smoker	62 (38.8)	32 (40.0)	30 (37.4)		
Current smoker	10 (6.2)	3 (3.8)	7 (8.8)		
AACVPR risk category				2.626	0.105
Low risk	130 (81.3)	69 (86.3)	61 (76.2)		
Moderate risk	30 (18.7)	11 (13.7)	19 (23.8)		
MI	124 (77.5)	59 (73.8)	65 (81.3)	1.29	0.344
PCI	108 (67.5)	55 (68.8)	53 (66.3)	0.114	0.866
CABG	51 (31.9)	25 (31.3)	26 (32.5)	0.029	1.0
Valve surgery	4 (2.5)	2 (2.5)	2 (2.5)		1.0^b^
Comorbidities					
Hypertension	136 (85.0)	67 (83.8)	69 (86.3)	0.196	0.658
Dyslipidemia	148 (92.5)	71 (88.8)	77 (96.3)	3.243	0.131
Diabetes	80 (50.0)	35 (43.8)	45 (56.3)	2.5	0.115
Peripheral vascular disease	2 (1.3)	2 (2.5)	0		0.497^b^
Transient ischemic attack	4 (2.5)	3 (3.8)	1 (1.3)		0.62^b^
Stroke	10 (6.3)	5 (6.3)	5 (6.3)	0.000	1.0
Family history of heart diseases	108 (67.5)	57 (71.3)	51 (63.7)	1.026	0.399
Medication					
Antiplatelet	160	80 (100.0)	80 (100.0)		
Nitrates	27 (16.9)	15 (18.8)	12 (15.0)	0.401	0.673
Beta‐blocker	129 (80.6)	64 (80.0)	65 (81.3)	0.04	1.0
ACE inhibitors/angiotensin/receptor blocker	71 (44.4)	36 (45.0)	35 (43.8)	0.025	1.0
Calcium channel blocker	49 (30.6)	28 (35.0)	21 (26.3)	1.441	0.303
Statin	160	80 (100.0)	80 (100.0)		

*Note:* Other data were analyzed using Chi‐square test (*X* ^2^).

Abbreviations: AACVPR = American Association of Cardiovascular and Pulmonary Rehabilitation, CABG = coronary artery bypass graft surgery, MI = myocardial infarction, PCI = percutaneous coronary intervention.

^a^ Independent *t*‐test.

^b^ Fisher's Exact Test.

^c^ Mann–Whitney *U* test.

^d^ IQR = Interquartile range.

### 3.2 Effects of TecHCR on Primary Outcome

The GEE model analysis (Table 3) revealed that participants in the intervention group demonstrated significantly greater improvement in exercise self‐efficacy compared with the control group at immediate post‐intervention (group‐by‐time interaction coefficient, *β* = 5.909, 95% CI [3.146, 8.672]; *p* < 0.001) and 24‐week post‐intervention (*β* = 8.506, 95% CI [4.951, 12.061]; *p* < 0.001). The effect sizes were nearly moderate (Hedges' *g* = 0.483, 95% CI [0.160, 0.807]) at T1 and moderate to large (Hedges' *g* = 0.755, 95% CI [0.375, 1.135]) at T2 when compared with center‐based CR (Table 3).

**TABLE 3:** Comparison of scores in outcome variables between TecHCR and control group across three time points using the generalized estimating equation (GEE) model.

Variables	Intervention	Control	Time effect		Group effect		Group‐by‐time effect	
			*ß* (95% CI)	*p*	*ß* (95% CI)	*p*	*ß* (95% CI)	*p*
Exercise self‐efficacy					0.301 (−4.285, 4.887)	0.898		
T0	41.91 ± 13.39	40.88 ± 14.57						
T1	50.84 ± 14.28	44.21 ± 13.15	3.138 (1.762, 4.514)	< 0.001**			5.909 (3.146, 8.672)	< 0.001**
T2	52.05 ± 11.62	42.37 ± 14.2	0.986 (−1.363, 3.335)	0.411			8.506 (4.951, 12.061)	< 0.001**
Health‐promoting behaviors					−1.212 (−8.782, 5.250)	0.622		
T0	131.34 ± 19.78	131.46 ± 23.79						
T1	148.6 ± 19.0	139.51 ± 20.6	7.893 (5.884, 9.794)	< 0.001**			9.058 (5.524, 12.591)	< 0.001**
T2	154.58 ± 18.99	138.33 ± 16.61	7.764 (4.552, 11.006)	< 0.001**			14.563 (8.809, 20.317)	< 0.001**
Exercise capacity					0.093 (−0.540, 0.726)	0.774		
T0	9.0 ± 2.24	8.6 ± 2.44						
T1	9.78 ± 2.1	9.32 ± 2.41	0.778 (0.462, 1.094)	< 0.001**			0.145 (−0.264, 0.553)	0.487
T2	9.89 ± 2.22	9.3 ± 2.09	0.743 (0.423, 1.062)	< 0.001**			0.342 (−0.111, 0.795)	0.139
Anxiety					−0.047 (−0.975, 0.881)	0.921		
T0	4.56 ± 2.95	4.4 ± 3.12						
T1	3.17 ± 1.9	4.39 ± 2.29	−0.036 (−0.420, 0.348)	0.855			−1.255 (−1.893, −0.616)	< 0.001*
T2	3.14 ± 2.38	4.41 ± 2.19	−0.063 (0.635, 0.510)	0.83			−1.145 (−1.975, −0.315)	0.007*
Depression					−0.470 (−1.428, 0.488)	0.336		
T0	3.8 ± 2.93	4.04 ± 3.16						
T1	2.95 ± 2.05	3.99 ± 2.34	−0.126 (−0.585, 0.333)	0.591			−0.599 (−1.301, 0.103)	0.094
T2	3.03 ± 2.37	3.8 ± 2.68	−0.366 (−0.980, 0.247)	0.242			−0.256 (−1.104, 0.591)	0.553
SBP (mmHg)					−0.487 (−4.275, 3.300)	0.801		
T0	134 ± 13.14	135 ± 12.28						
T1	133 ± 11.33	135 ± 1.72	−1.259 (−2.600, 0.083)	0.066			0.534 (−1.602, 2.670)	0.624
T2	131 ± 11.3	135 ± 9.96	−1.077 (−2.420, 0.266)	0.116			−1.475 (−4.254, 1.304)	0.298
DBP (mmHg)					−1.779 (−4.838, 1.280)	0.254		
T0	79 ± 8.99	81 ± 9.82						
T1	79 ± 8.47	80 ± 8.8	−0.931 (−1.929, 0.067)	0.068			0.884 (−0.685, 2.452)	0.27
T2	78 ± 6.70	80 ± 6.91	−0.344 (−1.547, 0.859)	0.575			−0.580 (−2.345, 1.185)	0.519
HDL‐C (mmol/L)					−0.008 (−0.062, 0.078)	0.818		
T0	0.97 ± 1.9	0.98 ± 0.27						
T1	1.04 ± 0.2	1.01 ± 0.27	0.029 (−0.005, 0.062)	0.096			0.031 (−0.020, 0.083)	0.231
T2	1.06 ± 0.18	1.03 ± 0.2	0.041 (0.000, 0.083)	0.051			0.025 (−0.037, 0.087)	0.429
LDL‐C (mmol/L)					−0.045 (−0.360, 0.269)	0.778		
T0	2.61 ± 1.11	2.60 ± 0.95						
T1	2.55 ± 0.96	2.59 ± 0.97	−0.02 (−0.083, 0.043)	0.537			−0.016 (−0.107, 0.074)	0.725
T2	2.56 ± 0.97	2.59 ± 0.80	0.006 (−0.107, 0.119)	0.918			−0.094 (−0.248, 0.062)	0.232
TG (mmol/L)					−0.090 (−0.347, 0.168)	0.495		
T0	1.84 ± 0.91	1.89 ± 0.86						
T1	1.83 ± 0.9	1.88 ± 0.86	−0.008 (−0.059, 0.042)	0.748			−0.038 (−0.135, 0.059)	0.437
T2	1.82 ± 0.81	1.88 ± 0.70	0.030 (−0.024, 0.085)	0.247			−0.029 (−0.120, 0.062)	0.527
TC (mmol/L)					−0.02 (−0.414, 0.374)	0.921		
T0	4.29 ± 1.32	4.19 ± 1.29						
T1	4.15 ± 1.25	4.17 ± 1.26	−0.055 (−0.111, 0.001)	0.05			−0.074 (−0.154, 0.006)	0.069
T2	4.15 ± 1.22	4.18 ± 1.16	−0.107 (−0.273, 0.059)	0.205			−0.140 (−0.319, 0.390)	0.126
HbA1c (%)					−0.150 (−0.358, 0.057)	0.155		
T0	6.14 ± 0.69	6.32 ± 0.84						
T1	6.13 ± 0.72	6.28 ± 0.87	−0.014 (0.089, 0.061)	0.712			−0.016 (−0.115, 0.083)	0.745
T2	6.13 ± 0.7	6.13 ± 0.7	0.036 (−0.058, 0.130)	0.45			−0.071 (−0.184, 0.042)	0.216
BMI (kg/m^2^)					0.384 (−0.522, 1.290)	0.407		
T0	25.64 ± 3.25	25.44 ± 3.35						
T1	25.47 ± 2.78	25.43 ± 3.26	0.016 (−0.108, 0.140)	0.795			−0.080 (−0.280, 0.120)	0.433
T2	25.22 ± 2.49	25.69 ± 2.6	0.162 (0.001, 0.324)	0.048			−0.188 (−0.408, 0.033)	0.095
Waist circumference (cm)					0.803 (−1.024, 2.630)	0.389		
T0	90.64 ± 8.26	90.60 ± 10.39						
T1	90.61 ± 7.97	90.51 ± 10.1	−0.027 (−0.198, 0.147)	0.754			−0.014 (−0.288, 0.315)	0.93
T2	90.27 ± 8.23	91.94 ± 9.27	0.064 (0.135, 0.263)	0.528			−0.138 (−0.321, 0.597)	0.555

*Note:* *p < 0.05, **p < 0.01.

Abbreviations: BMI = body mass index, DBP = diastolic blood pressure, HbA1c = glycated hemoglobin, HDL‐C = high‐density lipoprotein cholesterol, LDL‐C = low‐density lipoprotein cholesterol, SBP = systolic blood pressure, T0 = baseline, T1 = immediate post‐intervention, T2 = 24‐weeks post intervention, TC = total cholesterol, TG = triglyceride.

### 3.3 Effects of TecHCR on Secondary Outcomes

Referring to Table 3, the GEE model results revealed a significant group‐by‐time interaction effect in health‐promoting behaviors at immediate post‐intervention (*β* = 9.058, 95% CI [5.524, 12.591]; *p* < 0.001) and 24‐week post intervention (*β* = 14.563, 95% CI [8.809, 20.317]; *p* < 0.001). The intervention group also demonstrated a significantly greater reduction in mean scores of perceived anxiety, decreasing by −1.255 (95% CI [−1.893, −0.616]), *p <* 0.001 at T1 and −1.145 (95% CI [−1.975, −0.315]), *p =* 0.007 at T2 in addition to that of the control group. However, there were no significant differences in changes between groups in perceived depression and all cardiovascular risk factors among patients with CHD at T1 and T2 (all *p*‐values > 0.05).

The intervention group demonstrated a nearly moderate effect (*g* = 0.459, 95% CI: 0.135, 0.782) and a large effect (*g* = 0.904, 95% CI [0.519, 1.288]) in health‐promoting behaviors at T1 and T2, respectively, when compared with center‐based CR (Table 4). Meanwhile, the intervention group showed a moderate effect on perceived anxiety, with effect sizes of *g* = −0.579 (95% CI [0.905, −0.254]) at T1 and *g* = −0.553 (95% CI [−0.928, −0.179]) at T2.

**TABLE 4:** Effect sizes of significant outcome variables.

Outcome variables	Mean difference in change score (T1–T0)	Hedges' *g* effect size (95% CI)	Mean difference in change score (T2–T0)	Hedge's *g* effect size (95% CI)
Exercise self‐efficacy (ESE)	5.975	0.483 (0.16, 0.807)	8.7	0.755 (0.376, 1.135)
Health‐promoting behavior (HPLP II)	8.905	0.459 (0.135, 0.782)	14.497	0.904 (0.519, 1.288)
Anxiety (HADS‐A)	−1.214	−0.579 (−0.905, −0.254)	−1.145	−0.553 (−0.928, −0.179)

## 4 Discussion

Previous randomized controlled trials related to CR have been conducted in Asian countries, including Bangladesh, China, Egypt, India, Iran, demonstrating positive effects on patient outcomes (Mamataz et al. 2022). The present study is likely the first nurse‐led, technology‐assisted hybrid CR program in the Southeast Asia region, underpinned by HBM in its intervention development. The majority of participants (91.9%) in this study were aged 40 and over, which aligns with the findings of Khan et al. (2014), showing significant coronary artery disease presented in 76.5% of adults aged over 40 years (Khan et al. 2014). Similarly, the Global Burden of Disease dataset also reported that the incidence of coronary artery disease increases from the age of 40 (Khan et al. 2020). In addition, participants in this study were predominantly male (95%), similar to previous RCTs that reported more than 80% male participants in their CR programs (Maddison et al. 2019; Su and Yu 2021). Of all participants, 81.3% were categorized as low risk and 18.7% were moderate risk, based on AACVPR stratification, indicating both groups were less likely to experience adverse effects when exercising compared to the high‐risk group (Bhat et al. 2021). According to Thomas et al. (2019), the AACVPR recommends that patients with low‐to‐moderate risks have an option to participate in home‐based CR. The present study followed the recommendation by offering participants a hybrid CR that combined center‐based with home‐based CR. The most prevalent comorbidity among participants was dyslipidemia. Generally, Malaysians have a high prevalence of dyslipidemia, noting that a 15‐year longitudinal study reported 64% of Malaysians had elevated total cholesterol (Mohamed‐Yassin et al. 2021), and the National Health and Morbidity Survey 2015 showed that 64.8% of older adults in Malaysia were diagnosed with dyslipidemia (Sazlina et al. 2020).

The primary outcome in this study was exercise self‐efficacy. Exercising training is known as the fundamental component of CR, as highlighted by Stone et al. (2005), with the aims to facilitate patients in recovering from cardiac events and ideally foster a sustained increase in physical activity to maintain health benefits (Xing et al. 2020). The positive effect of TecHCR on exercise self‐efficacy was promising as Shea et al. (2023) highlighted that exercise self‐efficacy predicts exercise regimen adherence, which is a priority in CR to enhance patient outcomes. Our finding seems to differ from a previous study, where no significant difference in exercise self‐efficacy was observed between intervention (fitness watch, mobile app, push‐through and educational messages) and control groups (pedometer to record daily steps in a diary) (Park et al. 2021). Notably, each participant in TecHCR was equipped with a fitness watch (which served as the “cues to action” from HBM) to self‐monitor their exercise activities to enhance their self‐efficacy. During each individual weekly video call, participants received feedback and encouragement on their progress and were assisted by the nurse researcher in setting personal goals for the following 7 days to enhance their exercise performance. Problem‐solving has been identified as a strategy to boost self‐efficacy (Farrell et al. 2004). In this study, participants in the TecHCR group were assisted in problem‐solving to overcome perceived barriers to independently performing exercise training at home. Furthermore, empowering the participants by allowing them to take an active role in their exercise training (e.g., setting personal goals, self‐monitoring of own progress through wearable technology, and self‐monitoring of symptoms during exercise) could have also enhanced their exercise self‐efficacy.

According to Gostoli et al. (2016), a lack of active participation in health behaviors is associated with poor patient outcomes in CR. The TecHCR program demonstrated encouraging results in promoting health behaviors among patients with CHD. Similarly, a previous RCT found that the eHealth intervention group showed significantly improved health‐promoting behaviors compared to the control group (Su and Yu 2021). A web‐based educational support intervention program, underpinned by the HBM, reported a significant increase in the amount of physical exercise among patients with CHD (Wong et al. 2020). This suggests that integrating the HBM into the development of technology‐assisted CR interventions is useful for enhancing health behavior change. Additionally, a previous qualitative study suggests that a basis of behavior change support is built on a relationship of trust; while trust is cultivated by personalized and tailored support, where patients oversee their own health behavior change (Beishuizen et al. 2019). The nurse–patient relationship in this study was developed through weekly video calls, with the nurse researcher supporting participants in the intervention group in setting goals related to exercise and health behaviors. Moreover, a review by Strecher et al. (1986) suggested that self‐efficacy has a strong relationship with health behavior change and maintenance. In this study, there could be a potential influence of improved exercise self‐efficacy in behavior change related to the physical activity of the participants.

There were no significant differences between intervention and control groups in this study in exercise capacity at two time points. To date, limited evidence is available regarding exercise capacity as an outcome of a technology‐assisted hybrid CR program. According to Wessel et al. (2004), the normal exercise capacity is ≥ 7 METS. In our study, both groups demonstrated normal levels of exercise capacity at baseline. The intricate nature of cardiopulmonary functions may affect the exercise capacity, potentially explaining the failure to achieve a significant between‐group difference across time points. Other factors such as ventricular functional reserve and cardiac output are also associated with exercise capacity in CR (Hansen et al. 2005).

According to Sandesara et al. (2015), managing cardiovascular risk factors is one of the fundamental components of a modern CR. This was integrated in TecHCR through audio‐visual educational videos for patient education and facilitated discussions during the video calls to address concerns related to risk factors. Nevertheless, no significant differences in cardiovascular risk factors were found between groups, consistent with existing evidence (Pfaeffli Dale et al. 2015; Maddison et al. 2019). The reasons for the lack of significant effects on cardiovascular risk factors in these studies remain unclear.

In many instances, patients who have experienced a cardiac event have the highest priority on physical recovery (Murphy et al. 2016). But it is also important to address psychological well‐being because anxiety and depression have been linked to an increased risk of mortality in patients with heart disease (Celano et al. 2015). Although no significant effect was found on perceived depression in the TecHCR group when compared with the control group, the TecHCR group demonstrated a significant reduction in anxiety levels both at immediate post‐intervention and 24 weeks post‐intervention. In contrast, Pfaeffli Dale et al. (2015) found that patients who received fully automated daily short message service text messages and access to a supportive website had significantly higher anxiety levels. One of the possible reasons for the participants in the TecHCR group showing positive effects in perceived anxiety could be the trust built between the nurse researcher and participants over the study duration, particularly during the weekly video call as participants were able to discuss the barriers to health behavior change and get support in setting goals. Moreover, the fitness watch provided to participants served as a tool for home exercise training, with more confidence and less anxiety.

### 4.1 Implications for Practice and Research

Despite nonsignificant results in several outcomes when compared with the control group in this study, the TecHCR program provided similar effectiveness to a center‐based program, suggesting that it is a promising alternative to conventional, center‐based programs, not only during a pandemic but also in the long run, as the usage of technologies is growing rapidly across the globe. This study highlights the pivotal role nurses can play in improving patient outcomes in CR through a nurse‐led TecHCR program. By effectively incorporating exercise monitoring with a fitness tracker, delivering educational content via six audio‐visual videos, and facilitating weekly video calls over a 12‐week period, the program demonstrated success in enhancing exercise self‐efficacy, promoting health behaviors, and improving anxiety levels among patients with CHD. These findings suggest that nurses can assume a key role in secondary prevention care among patients with CHD. Additionally, building rapport with participants is necessary to foster active participation and cooperation, thereby optimizing patient outcomes from such interventions. In this study, HBM was used as a theoretical framework to guide the development of TecHCR. Nurses must possess comprehensive knowledge and skills related to CR and understanding of the HBM, which are crucial for facilitating patient engagement and maximizing the benefits of the program. By leveraging the constructs of HBM, nurses can enhance participants' awareness of the CR benefits and help them to overcome perceived CR barriers.

The TecHCR program, led by a nurse and supported by other healthcare professionals particularly physiotherapists and occupational therapists, used wearable technology like a fitness watch to enhance self‐efficacy (one of the constructs in HBM) through motivation and external cues provided by the researcher with exercise data captured by the fitness watch. This approach empowers patients to self‐manage their health and encourages behavioral change. Also, multidisciplinary collaboration can improve patient engagement and self‐care (Morley and Cashell 2017). Disseminating the findings of this study and sharing a standardized protocol with healthcare professionals and policymakers can increase awareness and facilitate the integration of technology‐assisted interventions into practice.

For future research, employing stratified sampling to include a larger population of female participants will ensure balanced gender representation within the study sample. This sampling method allows researchers to make statistical inferences from the sample to the broader population (Sharma 2017), providing a more thorough understanding of the intervention effects and revealing any gender‐specific differences in treatment outcomes. Although the findings in this study did not demonstrate significant effects in exercise capacity and cardiovascular risk factors, future research should consider including parameters such as left ventricular function and cardiac output to explore how these variables influence the exercise capacity of patients with CHD. In addition, future research should investigate the effects of pharmacotherapy (e.g., medications used, medication adherence) on cardiovascular risk factors in patients with CHD. Also, the TecHCR program was conducted in a capital city, which is an urban area where individuals are exposed to different technologies in their day‐to‐day lives. It is recommended to include patients from diverse settings, especially from rural areas where technology usage may be lacking, and infrastructure is underdeveloped. This will provide insights into different perceived needs and challenges in patients living in rural areas. For example, Sarawak is a state in Malaysia with an area of approximately 124,450 km^2^, but there is only one heart center that offers structured outpatient CR. More than half of the patients had to travel over 1 h and over 30 km to reach that center in Sarawak (Chai et al. 2020). Sugiharto et al. (2023) highlighted several barriers to participation in CR which also include environmental, logistical, and health system factors. It is not surprising that CR participation in a capital city was more than 50% (Chong et al. 2024), but a study that included five hospitals in Malaysia and a total of 402 patients showed only 29% of all patients were invited to participate in CR, and 17% of them completed more than half of the required sessions (McEvoy et al. 2024). The states where the five hospitals were located in Malaysia were not mentioned. Also, not all hospitals have an automatic referral to CR for all patients with CHD, unlike our study setting. It is worthwhile to include multiple settings in future research, as this may benefit patient outcomes from diverse geographical areas with different barriers related to environment, logistics, and health systems in CR.

### 4.2 Limitations

This study had several limitations that should be considered while interpreting its results. First, the participant recruitment was from a single center, which might introduce selection bias and affect population representativeness, thereby limiting the generalizability of the findings. Second, the relatively small number of female participants warrants caution in the interpretation of the results. Future studies should consider stratified randomization to ensure adequate representation of both genders. Involving multiple‐site settings will capture a representative sample of patients with CHD, which will improve the generalizability of the study findings. Third, self‐reported measures were employed to examine the changes in several outcomes (ESE, HPLP II, and HADS), which might be subjected to social desirability bias, a tendency for participants to respond in a way that is socially desirable. Furthermore, data collected through self‐reporting can either underestimate or overestimate the effects of an intervention (Althubaiti 2016). Nonetheless, this study also included several outcomes that were measured objectively, such as exercise capacity and cardiovascular risk factors, which were less likely to be affected by the Hawthorne effect. Fourth, due to the nature of the intervention, participants were unable to be blinded to their group assignments, potentially affecting the self‐reported outcomes. Lastly, this study examined the effects immediately after the intervention and 24 weeks post‐intervention, which did not constitute a long‐term period. Thus, conducting future research with long‐term follow‐up assessments will be necessary to gain a deeper understanding of the sustainability of the intervention effects.

## 5 Linking Evidence to Action

- Healthcare professionals and policymakers should advocate for the integration of hybrid technology‐assisted programs into standard care pathways to overcome barriers and improve accessibility.
- To enhance self‐efficacy, TecHCR used a fitness watch for self‐monitoring exercise. This watch served as “cues to action,” one of the Health Belief Model constructs, where heart rate and exercise data provided biofeedback that was used as an external trigger.
- Incorporating weekly video call follow‐ups by a healthcare professional to provide personalized feedback and assist with goal setting helps to build rapport between participants and the researcher.
- Researchers should ground intervention development in behavioral theories to promote better patient outcomes.
- Future iterations of hybrid CR should also integrate targeted strategies to address cardiovascular risk factors, as TecHCR was effective for exercise self‐efficacy, health‐promoting behaviors, and anxiety but not physiological outcomes.

## 6 Conclusion

This nurse‐led, 12‐week technology‐assisted intervention in a hybrid mode CR was developed drawing on evidence from a systematic review and meta‐analysis and results from a recent cross‐sectional study, while integrating the HBM framework. It is a relevant study especially because it was carried out during the COVID‐19 pandemic, when the integration of technology in CR rapidly advanced due to restrictions including limited in‐person exercise training sessions. This study provides compelling evidence that interventions utilizing technology and guided by the HBM can effectively enhance exercise self‐efficacy, encourage health behaviors, and improve anxiety levels. There is a need to further integrate and examine technology‐assisted interventions in both clinical practice and research to fully harness their potential and reach broader and more diverse populations. In addition, nurses as the largest sector in the global health workforce, are in a central position to actively participate in care delivery and address practice gaps to improve cardiac patient outcomes.

## Funding

The authors have nothing to report.

## Conflicts of Interest

The authors declare no conflicts of interest.

## Supporting information


**Data S1:** wvn70092‐sup‐0001‐Supinfo1.zip.

## Data Availability

The data that support the findings of this study are available on request from the corresponding author. The data are not publicly available due to privacy or ethical restrictions.
